# Melatonin promotes stomatal immunity by mediating phytohormone crosstalk in *Arabidopsis thaliana*

**DOI:** 10.3389/fpls.2026.1801537

**Published:** 2026-03-24

**Authors:** Yangyang Li, Tahmina Akter, Craig Dufresne, Yu Song, Jingkui Tian, Wei Zhu, Sixue Chen

**Affiliations:** 1 Institute of Applied Ecology, Chinese Academy of Sciences, Shenyang, China; 2 Department of Biology, University of Florida, Gainesville, FL, United States; 3 Genetics Institute, University of Florida, Gainesville, FL, United States; 4 Department of Biology, University of Mississippi, Oxford, MS, United States; 5 Thermo Fisher Scientific, West Palm Beach, FL, United States; 6 Hangzhou Institute of Medicine, Chinese Academy of Sciences, Hangzhou, China

**Keywords:** *Arabidopsis thaliana*, coronatine, melatonin, multi-omics, phytohormone crosstalk, stomatal immunity

## Abstract

Melatonin, a phytohormone, has drawn growing interest for its important role in improving plant tolerance to biotic and abiotic stresses. However, its specific function and underlying mechanisms in stomatal immunity remain to be fully elucidated. In this study, we investigated the role of melatonin in mitigating bacterial invasion of *Arabidopsis thaliana* by modulating stomatal immune responses. To achieve this, melatonin biosynthesis and receptor mutants were employed to monitor guard cell responses to *Pseudomonas syringae*. Furthermore, single-cell type metabolomics and multi-omics analyses were integrated to dissect the signaling pathways involved. The results demonstrated that melatonin is essential for maintaining stomatal closure during bacterial infection. Single-cell metabolomics revealed that melatonin decreases jasmonic acid (JA) biosynthesis, thereby alleviating the antagonistic interaction with salicylic acid (SA) and positively regulating resistance to bacterial infections. Moreover, multi-omics data support melatonin-mediated crosstalk among JA/coronatine, SA-Non-Pathogenesis-Related 1 (NPR1), and reactive oxygen species/nitric oxide (ROS/NO) in stomatal immunity. This study reveals a sophisticated melatonin-regulated phytohormone crosstalk that orchestrates stomatal immunity against bacterial pathogens. These findings provide novel insights into the integration of melatonin signaling with systemic defense responses in plants.

## 1 Introduction

Melatonin (N‐acetyl‐5‐methoxytryptamine) was first isolated from the bovine pineal gland in animals (Lerner et al., 1958). Early studies described its role in detoxifying free radicals in both animals and plants (Manchester et al., 2015; Tan et al., 2015; Taboada et al., 2023). Melatonin plays an important role in plant resistance to abiotic and biotic stresses by regulating cellular redox homeostasis and hormone levels/signaling (Arnao et al., 2022; Taboada et al., 2023; Michalek et al., 2025). In the early stage of pathogen invasion, formation of a melatonin–reactive oxygen species (ROS)–reactive nitrogen species (RNS) feedforward loop maximizes disease resistance, and melatonin receptor–mitogen-activated protein kinase (MAPK) cascade forms a typical melatonin signaling pathway to facilitate stomatal closure and limit pathogen entry (Zhao et al., 2021; Jiang et al., 2025). This stomatal immunity is the first line of defense against pathogen invasion (Melotto et al., 2006). Melatonin regulation of stomatal immunity involves an active mechanism. It may be a downstream regulator of Flagellin Sensing 2 (FLS2) and Brassinosteroid insensitive 1-Associated Kinase 1 (BAK1) immune receptors (Yang et al., 2021; Khan et al., 2023), which transmit flagellin peptide of N-terminal 22 amino acids (flg22)-mediated stomatal immunity (Su et al., 2017). The first phytomelatonin receptor 1 (PMTR1) was identified in *Arabidopsis thaliana*, and it regulates two downstream signaling pathways in stomatal immunity. One is via MAPK activation, and the other is via heterotrimeric GTP-binding protein Ga subunit (GPA1) (Wei et al., 2018). Both pathways involve activation of nicotinamide adenine dinucleotide phosphate (NADPH) oxidase and ROS production to induce stomatal closure. Although the interactions between PMTR1 and GPA1 are necessary for melatonin- and flg22-induced stomatal closure, the activation of MAPKs is contingent upon PMTR1 not GPA1 (Yang et al., 2021; Khan et al., 2023).

In countering stomatal immunity, pathogens evolved a phytotoxin coronatine (COR), which inhibits pattern-triggered immunity (PTI) through hijacking the jasmonic acid (JA) signaling pathway and reopens stomata (Zhang et al., 2015; Velásquez et al., 2017). Plants then activate effector-triggered immunity (ETI) as a secondary immune response to trigger a hypersensitive response (HR). Melatonin has been hypothesized to act as an ETI enhancer and a PTI reinforcer, boosting ROS production to close stomata (Tanaka et al., 2015; Zhang et al., 2015). How melatonin plays a role in regulating PTI and ETI of stomatal defense requires further investigation.

Melatonin regulation may be upstream of various hormone actions in pathogen invasion (Hardeland, 2019). It is known that the interaction of salicylic acid (SA) and JA is the prevailing mechanism underlying stomatal movement regulation (Zeng et al., 2011). SA is rapidly induced during early events in stomatal immunity and can be enhanced by melatonin to activate pattern recognition receptor (PRR) genes, preventing bacteria from entering the plant cell (Hernández-Ruiz and Arnao, 2018). COR, a structural mimic of JA-isoleucine (JA-Ile), hijacks the JA signaling pathway by binding tightly to coronatine insensitive 1 (COI1) and removing transcriptional inhibition by JASMONATE-ZIM DOMAIN (JAZ) proteins (Wang et al., 2020). Thus, COR antagonizes SA and PTI to reopen the stomata (Gimenez-Ibanez et al., 2017; Uddin et al., 2022). Exogenous application of melatonin induces the accumulation of SA and ethylene (ET), leading to increased expression of resistance genes (Lee et al., 2014). Furthermore, activation of abscisic acid (ABA) and the inhibition of ET by melatonin are incorporated into the regulatory system of melatonin’s function in stomatal immunity (Kou et al., 2021; Waseem et al., 2025). However, how these hormones crosstalk during the initial stomatal closure and then being pried to reopen is not known.

In our study, stomatal guard cell interactions with *Pseudomonas syringae pv* tomato (*Pst*) DC3000 and coronatine-deficient *Pst cor^−^* were used to investigate the active role of melatonin and its crosstalk with other hormones in stomatal immunity. The reverse genetics approach involving the biosynthesis and signaling of melatonin and JA was applied. The melatonin-PMTR1 regulatory network was validated through multi-omics analysis and with JA/SA-related mutants in plant innate immunity, providing supporting information on phytohormone crosstalk in stomatal immunity.

## 2 Materials and methods

### 2.1 Plant materials

*A. thaliana* seeds for wild-type (WT, Columbia ecotype), JA, and SA mutants (*COI1* knockout: *coi1-12*, *EDS1* knockout: *eds1*, and *NahG transgenic line*: *NahG1*) were provided by Dr. Zhonglin Mou, Department of Microbiology and Cell Science, University of Florida, USA. The seeds of *JAZ7* overexpression mutant (*jazOE*) (Yan et al., 2014) and the melatonin receptor mutant (*pmtr1*) (Wei et al., 2018) were obtained from the *Arabidopsis* Biological Resource Center (Columbus, OH, USA). The triple mutant (*triple*) of melatonin biosynthesis was generated by crossing *snat1* (salk_020577), *asmt1* (salk_067718), and *comt1* (CS25167) (Byeon et al., 2014; Li et al., 2023b). The triple mutant with homozygous alleles was used. The *Arabidopsis* seeds were sterilized with 30% bleach for 10 min, washed with sterilized water, and placed on a half-strength Murashige and Skoog (MS) medium in darkness at 4°C for 2 days (Davis et al., 2009). After germination, the seedlings were grown at 140 µmol m^−2^ s^−1^ light intensity and 65% relative humidity with an 8-h light/16-h dark cycle for 4 weeks. The leaves were collected and used in this study.

### 2.2 Guard cell isolation

Enriched guard cell samples were prepared using a tape-peel method (Geng et al., 2016). Briefly, clear scotch tape (3M, St Paul, MN, USA) was attached to the abaxial epidermis of the *A. thaliana* leaves and separated quickly. The peels were transferred to an enzyme solution (0.7% Calbiochem Cellulysin R10, 0.025% Macerozyme R10, 0.25% BSA, and Y23 Pectolyase) to digest epidermal and mesophyll cells for 7 min at 22 ± 1°C. Then, the peels were washed three times with distilled water and incubated in an opening buffer (10 mM KCl, 50 mM CaCl_2_, 10 mM MES-KOH, adjusted to pH 6.15 with 1 M KOH) for 1 h under light. The peels were stained with neutral red (NR) and fluorescein diacetate (FDA) for guard cell purity and viability (at least 93%) following a previous method (Kang et al., 2021).

### 2.3 Bacterial assay

*Pst* DC3000 and a COR-deficient mutants of *Pst* DC3118 (*Pst cor^−^*) were kindly provided by Dr. Gregory Martin from the Boyce Thompson Institute for Plant Research (Ithaca, NY, USA), and they were grown in King’s B agar medium with antibiotics (0.1% (v:v) kanamycin and 0.05% rifampicin for *Pst* DC3000; 0.1% ampicillin and 0.05% rifampicin for *Pst cor^−^*) (Kang et al., 2021). A single colony was transferred to a sterile culture tube containing 10 mL of King’s B liquid medium and incubated in a shaker at 225 rpm and 28 °C overnight. Then, *Pst* and *Pst cor^−^* containing culture was centrifuged and resuspended in 10 mM MgCl_2_ to an OD_600_ of 0.2 for bacterial treatment. The mock treatment was 10 mM MgCl_2_.

The mock and bacterial suspensions were sprayed separately on the surface of the *A. thaliana* leaves evenly. After 2 days, infected leaves were separated and rinsed with sterile water to conduct a bacterial assay. A sterilized punch was used to obtain three leaf discs (diameter = 1 cm) from each infected leaf, which were placed in 500 μL sterilized water. The discs were ground with a plastic pestle and diluted 1,000 times (1:1,000). The bacterial solution was spread onto the agar plates and incubated at 28°C for 2 days for bacterial colony counting. This experiment was repeated three times with three replicates each time. The bacterial counts from nine replicates were used to calculate the mean and standard error.

### 2.4 Stomatal movement assay

After recovery in the opening buffer under light, the guard cell peels were pretreated with 1 μM melatonin before bacterial treatment. Then, the guard cell peels were transferred to the *Pst* and *Pst cor*− bacterial solution (OD_600_ = 0.2) for bacterial treatment. A stomatal movement assay was performed using a ZEISS fluorescence microscope (Axio Observer A1, Bonn, Germany) at 0, 1, and 3 h post-inoculation (hpi). Five replicates were conducted for each treatment at each timepoint. ImageJ software (National Institutes of Health, Maryland, USA) was used for measuring stomatal apertures. The stomatal aperture index was calculated by dividing the width of stomate by its length.

### 2.5 Untargeted metabolomics of enriched stomatal guard cells

Metabolites from the enriched guard cells were extracted as described (David et al., 2021). Untargeted metabolomic profiling was performed on a Vanquish Horizon UPLC system (Thermo Scientific, San Jose, CA, USA) coupled to an Orbitrap Fusion Tribrid mass spectrometer as previously described (Li et al., 2023b). In brief, a reverse-phase C18 column (Thermo Scientific, Accucore C18, 100×2.1 mm, 2.6 μm) was used for metabolite separation at a flow rate of 0.45 mL min^−1^ (55°C) with 0.1% formic acid in water as solvent A and 0.1% formic acid in acetonitrile as solvent B. The gradient was applied: 0-0.5 min, 0.1% solvent B; 0.5-6.0 min, 0.1%-40% solvent B; 6.0-7.5 min, 40%-98% solvent B; 7.5-8.5 min, 98% solvent B. Mass spectrometry (MS) analysis was performed under the following conditions: sheath, auxiliary, and sweep gas flows were set to 50, 10, and 1 arbitrary unit, respectively. The ion transfer tube and vaporizer temperatures were maintained at 325°C and 350°C, respectively. Spray voltages were +3,500 V in positive and −2500 V in negative mode, with a full-scan mass range of 55–550 m/z. For metabolite identification, data-dependent MS^n^ analysis was carried out in both polarity modes. Spectra were acquired with an Orbitrap resolution of 7.5 K, a maximum injection time of 20 ms, and an automatic gain control (AGC) target of 50,000. Raw data files were analyzed using Compound Discoverer 3.0 (Thermo Scientific, San Jose, CA, USA), and metabolite identification was performed against the mzCloud, KEGG, ChemSpider, and PubChem databases.

### 2.6 Proteomics of enriched stomatal guard cells

Proteins from 50 enriched guard cell peels were extracted in 80% acetone containing 10% trichloroacetic acid and 0.07% 2-mercaptoethanol. After vortexing with glass beads and sonicating for 5 min, the solution was incubated at −20°C overnight followed by centrifugation at 9,000×g for 20 min at 4°C. The supernatant was discarded, and the pellet was washed twice with 0.07% 2-mercaptoethanol in acetone. The pellet was dried using a SpeedVac concentrator and resuspended in a solubilization solution (7 M urea, 2 M thiourea, 5% CHAPS, and 2 mM tributylphosphine) by vortexing at room temperature for 1 h. The solution was centrifuged at 20,000×g for 20 min at 4°C. Then, the supernatant was collected, and the protein concentration was determined using a Bradford assay with bovine serum albumin as the standard. Protein digestion and LC-MS/MS data acquisition were performed as previously described (Zhu et al., 2021a). Data analysis was performed by using Proteome Discoverer™ 2.4 (Thermo Scientific, Bremen, Germany), and the SEQUEST algorithm was used to process the raw files. The differentially expressed proteins (DEP) were selected based on the criteria of fold change > 2 or < 0.5 with *p*-value < 0.05. The significant DEPs were used for functional enrichment analysis by Kyoto Encyclopedia of Genes and Genomes (KEGG) and Gene Ontology (GO).

### 2.7 RNA extraction and quantitative analysis

Total RNA extraction was conducted as previously described (Li et al., 2023a). Real-time quantitative PCR (qRT-PCR) was performed using the SYBR Premix Ex Taq™ Kit (Applied Biosystems, CA, USA). Three biological and three technical replicates were carried out. The relative gene expression was calculated by using the Ct (2^−ΔΔct^) method (Livak and Schmittgen, 2001), and **Supplementary Table 1** contains the list of the primers used in this study.

### 2.8 Data analysis

All statistical analysis were conducted using SPSS version 8.0 (SAS Institute, Cary, NC, USA), including partial least squares discriminant analysis (PLS-DA). Statistical significance was evaluated by Student’s t-test when only two groups were compared and one-way ANOVA with Tukey’s test when multiple groups were compared. A p-value from an unpaired Student’s *t*-test less than 0.05 and/or 0.01 was considered as statistically significance. All figures were created using GraphPad Prism 10 (Boston, MA, USA). Web Gene Ontology Annotation Plot (WEGO) 2.0 has been used for visualizing the gene ontology annotations (Ye et al., 2006, 2018). WEGO is available for free at http://wego.genomics.org.cn.

## 3 Results

### 3.1 Melatonin regulates stomatal immunity against *Pseudomonas syringae* infection

**Figure 1:**
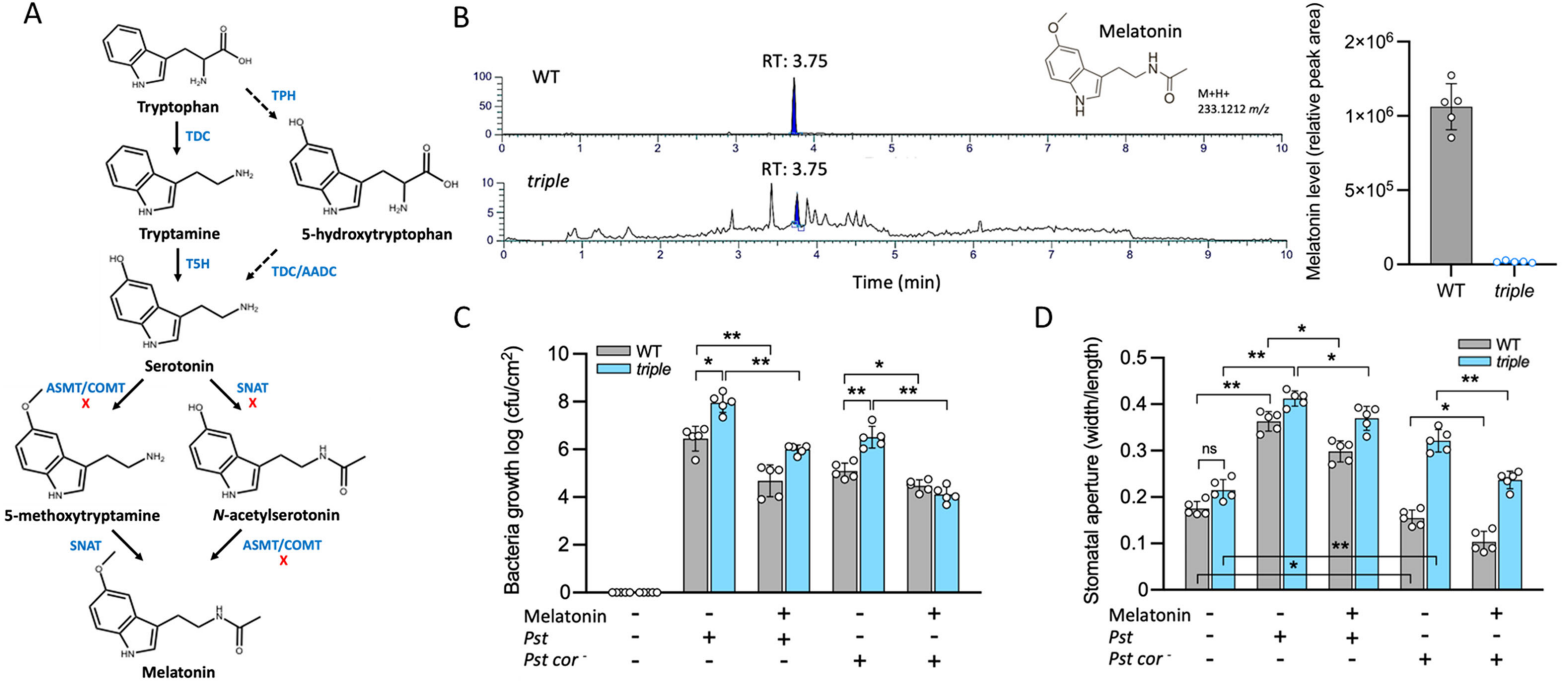
Effects of melatonin on stomatal immune responses to *Pseudomonas syringae* pv tomato (*Pst*) in wild type (WT) and a melatonin mutant (*triple*). **(A)** Melatonin biosynthesis pathway in plants. Abbreviations: TPH, tryptophan hydroxylase; TDC, tryptophan decarboxylase; T5H, tryptamine 5-hydroxylase; AADC, aromatic amino acid decarboxylase; SNAT, serotonin acetyltransferase; ASMT, N-acetylserotonin methyltransferase; COMT, caffeic acid o-methyltransferase. **(B)** Melatonin contents in the WT and *triple* mutant. **(C)** Assessment of bacterial growth under *Pst* and *Pst cor−* treatments in the presence and absence of melatonin. “+” and “−“ denote presence and absence, respectively (n=9). **(D)** Effect of melatonin on stomatal movement under *Pst* and *Pst cor−* treatments with and without melatonin. Values of stomatal assay were shown in the stomatal aperture index (width/length) as mean ± SE (n=15), and “*” and “**” indicate *p*-values <0.05 and <0.01, respectively, by Student’s t test.

To determine the role of melatonin in stomatal immunity, a triple mutant (*aka triple*) of N-acetyl transferase (*SNAT*), N-acetylserotonin methyltransferase (*ASMT*), and N-acetylserotonin (*COMT*) was used (Weng et al., 2011; Li et al., 2023b). The three enzymes catalyze the biosynthesis of melatonin from tryptophan (Arnao and Hernández-Ruiz, 2006; Park and Back, 2012) (**Figure 1A**). There was a 105-fold decrease of melatonin in the *triple* compared with WT (**Figure 1B**). The *triple* plants with T-DNA inserted into the intron region of each gene showed morphological differences at different growth stages (**Supplementary Figure 1A**). The seedlings grew more slowly than WT, accompanied by smaller leaves, delayed bolting, and reduced reproductive capacity (smaller flowers and pods) (**Supplementary Figure 1B**). When *Pst* and *Pst cor^−^* were spray inoculated, the *triple* and WT plants showed differential responses in bacterial growth and stomatal movement (**Figures 1C, D**), suggesting that melatonin may play an important role in this defense process. Due to the lack of COR, the pathogenicity of *Pst cor^−^* was slightly weaker than that of *Pst* (**Supplementary Figure 1C**). The *triple* leaves contained higher bacterial titers compared with WT, and bacterial colonization was inhibited by exogenous melatonin (**Figure 1C**). Further analysis of stomatal conductance revealed that the stomatal aperture in the *triple* was larger than that of WT, but the difference was not significant under normal conditions. However, stomatal conductance was significantly increased by threefold compared with WT after 2 days *of Pst* inoculation, and this mutant effect was reversed by exogenous melatonin addition. The treatment with *Pst cor^−^* showed an interesting difference in stomatal movement between the *triple* and WT, with stomatal aperture decreased in WT but increased in the *triple* (**Figure 1D**). These data indicate that melatonin is involved in the plant’s early defense by enhancing stomatal immune response. An exploration of the mechanisms underlying the melatonin and COR effects may provide interesting insight.

### 3.2 Melatonin prevents stomatal reopening eminently under bacterial invasion

**Figure 2:**
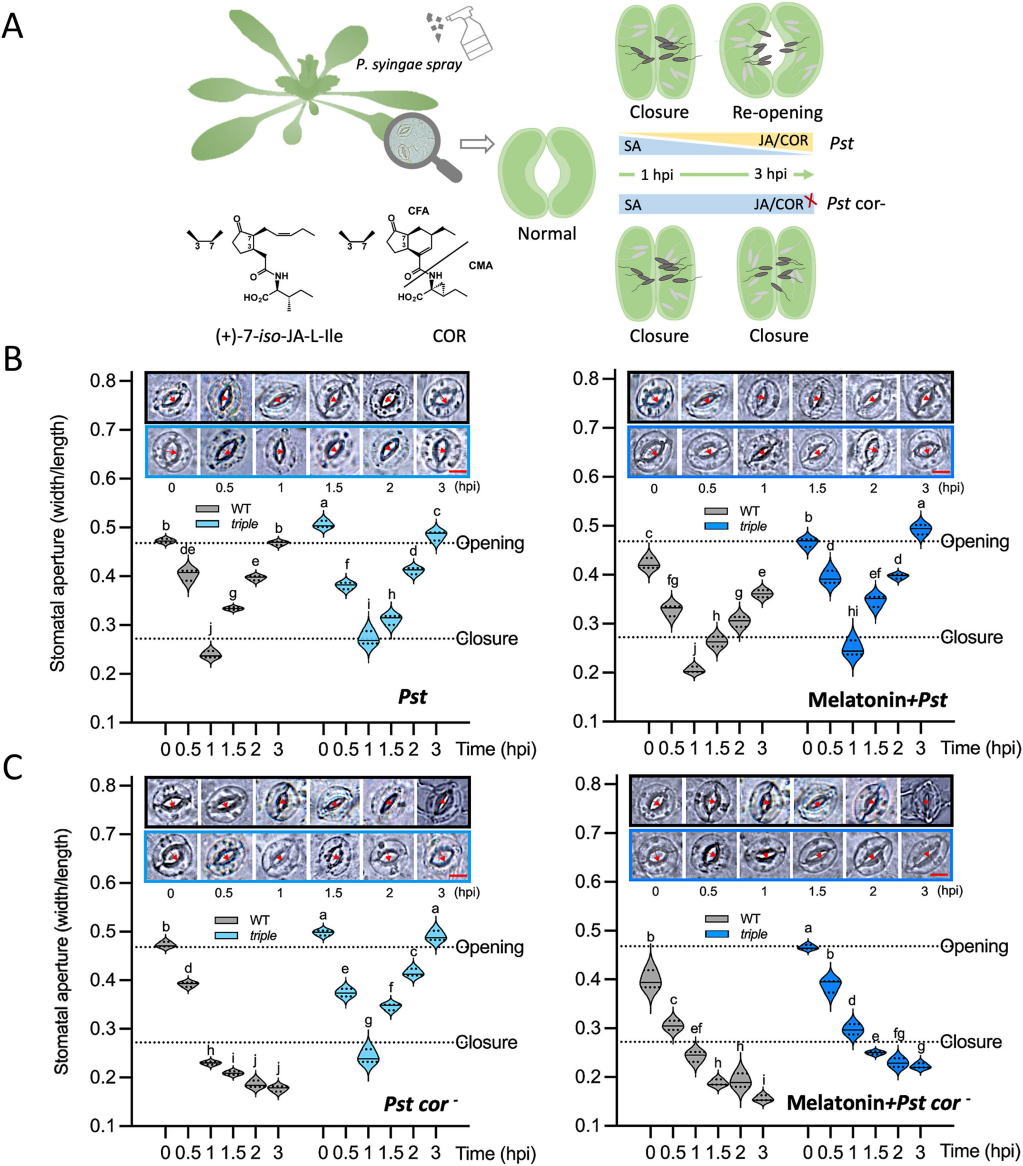
Stomatal movement phenotypes under spray infection of *Pst, Pseudomonas syringae* pv tomato with or without coronatine (COR) in wild type (WT) and a melatonin mutant (*triple*). **(A)** Simplified diagram depicting coordination of salicylic acid (SA); JA, jasmonic acid, and COR in stomatal movement in response to *Pst* and *Pst* cor*^−^* treatments. As an initial response upon pathogen perception, SA increases to close the stomata. COR as a structural mimic of JA-isoleucine (JA-Ile) suppresses stomatal defense and reopens the stomata to facilitate pathogen entry. COR deficient *Pst* is unable to reopen the stomata. **(B)** Stomatal movement in the presence and absence of melatonin under *Pst* invasion. **(C)** Stomatal movement in the presence and absence of melatonin under *Pst cor^−^* invasion. Five replicates of each treatment at each time point, and a total of three independent experiments were conducted. The bar indicates 5 µm.

Stomata closed within an hour post infection (hpi) when plasma membrane receptors (e.g., FLS2) sense *Pst*. At approximately 3–4 hpi, stomata are pried open by COR from the *Pst* (Allu et al., 2016; Lajeunesse et al., 2023; Liu et al., 2024). **Figure 2A** shows a simplified diagram of stomatal immunity summarizing the underlying interactions among SA, JA, and COR. COR is a structural mimic of JA-Ile that represses SA levels by inducing COI1–JAZ interaction and mediating JAZ degradation. Based on the pattern of stomatal closure and reopening, WT and *triple* seedlings were treated by *Pst* and *Pst cor^−^* to assess melatonin functions. The results revealed that stomata were closed within 1 hpi and reopened after 3 hpi in WT under *Pst* treatment. The *triple* mutant lacking melatonin showed a larger aperture than WT during stomatal closure and reopening. The stomatal aperture decreased significantly by the external melatonin addition, both in the processes of closing and reopening (**Figure 2B**). It appears that melatonin plays a positive role in closing stomata. However, the stomatal movement showed a different mode under *Pst cor^−^* treatment (**Figure 2C**). Without COR, the stomata were continuously closed in WT but reopened in the *triple*, and this reopening could be blocked by the addition of exogenous melatonin. It was inferred that melatonin inhibits stomatal reopening, and this process clearly succumbed to the COR-regulated pathways, but not to JA pathways (**Figure 2A**).

### 3.3 Metabolomics of guard cells after bacterial infection of WT and melatonin mutant

**Figure 3:**
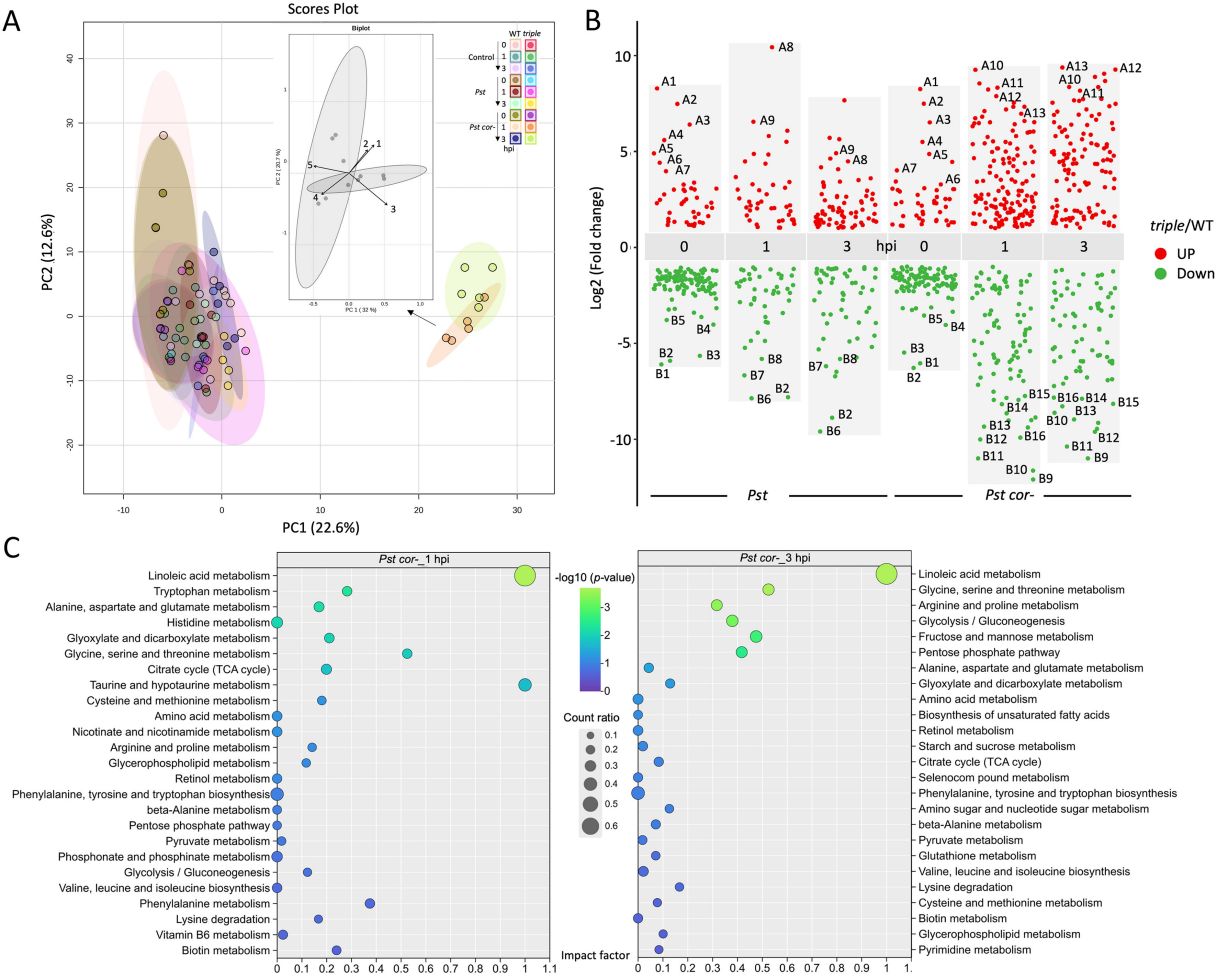
Metabolic profiling of guard cells at different timepoints after invasion of *Pseudomonas syringae* pv tomato (*Pst*) with or without coronatine (COR) in wild type (WT) and a melatonin mutant (*triple*). **(A)** Principal component analysis (PCA) of 18 groups of conditions (Control, *Pst*, and *Pst* cor*^−^*; 0, 1, and 3 h postinfection (hpi); WT and *triple*). Inset: partial least squares-discriminant analysis (PLS-DA) of *Pst cor^−^* 1 and 3 hpi, as detailed in **Supplementary Table 2** (point 1: histidine; point 2: arginine; point 3: 4-dodecylbenzenesulfonic acid; point 4: 3-methyladenine; point 5: (11E,15Z)-9,10,13-trihydroxyoctadeca-11,15-dienoic acid). **(B)** Volcano plot showing significantly changed metabolites (SCMs) among the comparison groups. Red and green dots indicate significantly increased and decreased metabolites, respectively (*p*-value<0.05, fold change cutoff was set to <0.5 or >2). Gray dots indicate non-significantly changed metabolites. The SCMs include A1: glutamine; A2: lysine; A3: boschnaloside; A4: N-alpha-L-ornithine; A5: diethylamine; A6: 2,3-pyridinedicarboxylic acid; A7: 4-imidazoleacrylic acid; A8: 12-oxo-phytodienoic acid; A9: citric acid; A10: 2,3-dihydroxypropyl 12-methyltridecanoate; A11: 5-(3-cyclohexylprop-1-ynyl)nicotinic acid; A12: anisole; A13: 13-HpODE; B1: 6,7-dihydro-3H-purine-2,6-diamine; B2: melatonin; B3: PC; B4: ectocarpene; B5: L-phenylalanine; B6: 2-amino-1,3,4-octadecanetrio; B7: melibiose; B8: orotic acid; B9: 4-hydroxyphenylpyruvate; B10: 2-hydroxy-6-[(8Z,11Z)-pentadeca-8,11,14-trien-1-yl]benzoic). **(C)** Pathway enrichment analysis of the SCMs between *triple* and WT groups in response to *Pst cor^−^*. The x-axis indicates the pathway impact factor, reflecting the topological importance of altered metabolites within each pathway, whereas the y-axis lists the corresponding metabolic pathways. The circle color denotes statistical significance expressed as −log10(p-value) (blue to green gradient, with higher values indicating greater significance), and circle size represents the count ratio, i.e., the proportion of SCMs relative to the total number of metabolites annotated in each pathway.

To determine potential melatonin functions in the stomatal immune responses, single-cell-type metabolomics was conducted to profile metabolite changes in WT and the *triple* mutant after *Pst* and *Pst cor^−^* treatment. As shown in the PLS-DA results, two *triple mutant* groups under *Pst cor^−^* treatments (1 and 3 hpi) were separated far away from the other groups, highlighting significantly different responses with extremely low melatonin (**Figure 3A**). It also indicates that without COR, the effect of melatonin deficiency becomes obvious. A total of 498 unique metabolites in guard cells were identified using MS/MS database matching, quantified, and categorized using KEGG and ChEBI annotations (Sumner et al., 2007) (**Supplementary Table 2**). Significantly changed metabolites (SCMs) were selected by comparing between the WT and *triple* mutant under different bacterial treatments (*p*-value < 0.05). Compared with the WT, a total of 44 and 57 SCMs decreased in the *triple* under 1 and 3 hpi *Pst* treatment whereas 43 and 68 were increased, respectively. Under the treatment of *Pst cor*^−^, 88 and 76 SCMs decreased in the *triple* under 1 and 3 hpi, whereas 142 and 119 were increased at 1 and 3 hpi, respectively (**Figure 3B**). Of the associated pathways related to melatonin (WT *vs. triple*) in stomatal response to bacteria, the top 25 SCM-related pathways showed different metabolic processes at 1 and 3 hpi of *Pst cor^−^* treatment (**Figure 3C**). At *Pst cor^−^* 3 hpi, gluconeogenesis, pentose phosphate pathway, and metabolism of glycine/serine/threonine were increased compared with those at *Pst cor^−^* 1 hpi. In addition to the citrate/TCA cycle, other amino acid and carboxylic acid pathways were decreased at 3 hpi (e.g., alanine, aspartate, glutamate, cysteine, methionine, glyoxylate, dicarboxylate, and glycerophospholipid metabolism). Notably, linolenic acid metabolism showed a significant increase from 1 to 3 hpi in the *Pst cor*^−^ treatment. Linolenic acid is an essential omega-3 fatty acid that functions as a precursor in the biosynthesis of JA (Li et al., 2016; Hao et al., 2025). The positive closing function of melatonin was evidenced in the WT, whereas contrasting phenotypes were observed in the *triple* under *Pst cor^−^* treatment (**Figure 2C**). In WT with melatonin and *Pst*, the biosynthesis of linolenic acid decreased, resulting in a decrease of JA. While in the *triple* with little melatonin, linolenic acid and JA increased, which may stimulate stomata reopening (**Figure 2B**, **Supplementary Table 2**). This result indicates that melatonin may regulate essential crosstalk of phytohormones involved in stomatal immunity, primarily decreasing JA biosynthesis and signaling.

### 3.4 Guard cell proteomics after bacterial infection of WT and melatonin mutant

**Figure 4:**
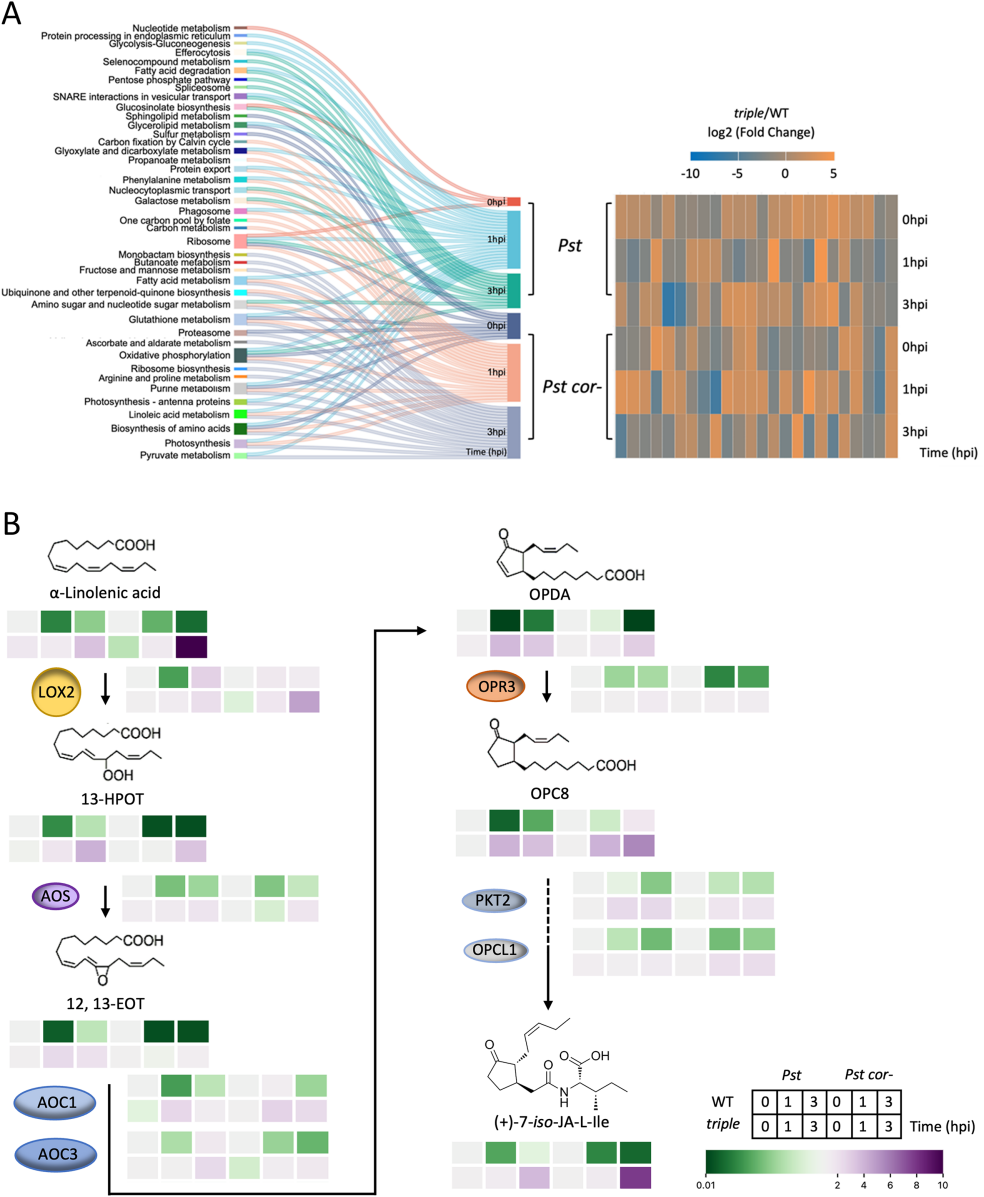
Differentially abundant proteins in guard cells and integrative metabolomics/proteomics of linoleic acid metabolism in stomatal immunity against bacterial invasion. **(A)** Differentially abundant proteins (DAPs) identified across the comparison groups (left panel, based on the KEGG database). The heatmap (right panel) represents the overlapped DAPs among six treatments between *tripe* and WT groups, which is detailed in **Supplementary Figure 3** (*p*-value<0.05, fold change cutoff was set to <0.5 or >2). **(B)** Schematic illustration of the jasmonic acid (JA) biosynthesis from α-linolenic acid to the bioactive (+)-7-iso-jasmonoyl-L-isoleucine (JA-Ile). The pathway initiates in the chloroplast with α-linolenic acid, which is oxygenated by LOX2 to form 13-HPOT. AOS converts 13-HPOT into the unstable epoxide 12,13-EOT, which is subsequently cyclized by AOC1 and AOC3 to produce OPDA. OPDA is transported to the peroxisome, where it is reduced by OPR3 to yield OPC8. OPC8 then undergoes β-oxidation, requiring OPCL1 for CoA activation and PKT2 for chain shortening, ultimately producing JA, which is conjugated to Ile to form the JA-Ile. The bars adjacent to each metabolite or enzyme indicate relative levels in wild type (WT) and in *triple* following treatment with *Pseudomonas syringae* pv. Tomato (*Pst*) or *Pst cor−* at 0, 1, and 3 h post infection (hpi). Color intensity represents relative abundance (green = low, purple = high; scale shown). Abbreviations: 13-HPOT, 13(S)-hydroperoxy-9(Z),11(E),15(Z)-octadecatrienoic acid; 12, 13-EOT, 12,13-epoxy-9(Z),11,15(Z)-octadecatrienoic acid; OPDA, 12-oxo-phytodienoic acid; OPC8, 8-oxo-phytodienoyl-coa; LOX, lipoxygenase; AOS, allene oxide synthase; AOC, allene oxide cyclase; OPR, 12-oxophytodienoate reductase; PKT2, phosphate and keto acid toxicity 2; OPCL, 12-oxophytodienoic acid-CoA ligase.

Guard cell proteomics was conducted to facilitate the understanding of phytohormone cross talk in stomatal immune responses of WT and *triple*. A total of 3,294 proteins were identified with at least two unique peptides (**Supplementary Table 3**). Compared between the *triple* and WT groups, a total of 73 and 75 DAPs were found under *Pst* and *Pst cor^−^* treatments, respectively (**Supplementary Figure 2A**). WEGO functional classification of the DAPs between *the triple* and WT grouped them into cellular component, biological process, and molecular function categories (**Supplementary Figure 2B**). With low melatonin, the DAPs were associated with chloroplast stroma, vacuoles, and thylakoids. Under *Pst* treatment, various metabolic processes of carboxylic acids, sulfur compounds, aldehydes, and photosynthesis were perturbed, whereas under *Pst cor^−^* treatment, differences in peroxisome and oxidoreductase activity were observed (**Supplementary Table 2**). Based on KEGG and ChEBI annotations, the DAPs were classified into 40 categories (**Figure 4A**). Integrative analysis of guard cell proteomics and metabolomics revealed potential pathways involved in melatonin regulation. They are metabolism of nitric oxide (NO) and ROS, JA- and SA-related defense pathways in stomatal immunity between the *triple* and WT (**Figure 4A**). Clearly, melatonin plays essential regulatory roles in stomatal immunity by participating in complex signaling pathways that involve different phytohormones and other signaling/metabolic factors.

### 3.5 Effects of melatonin on linolenic acid metabolism, and JA and SA crosstalk in stomatal immunity

**Figure 5:**
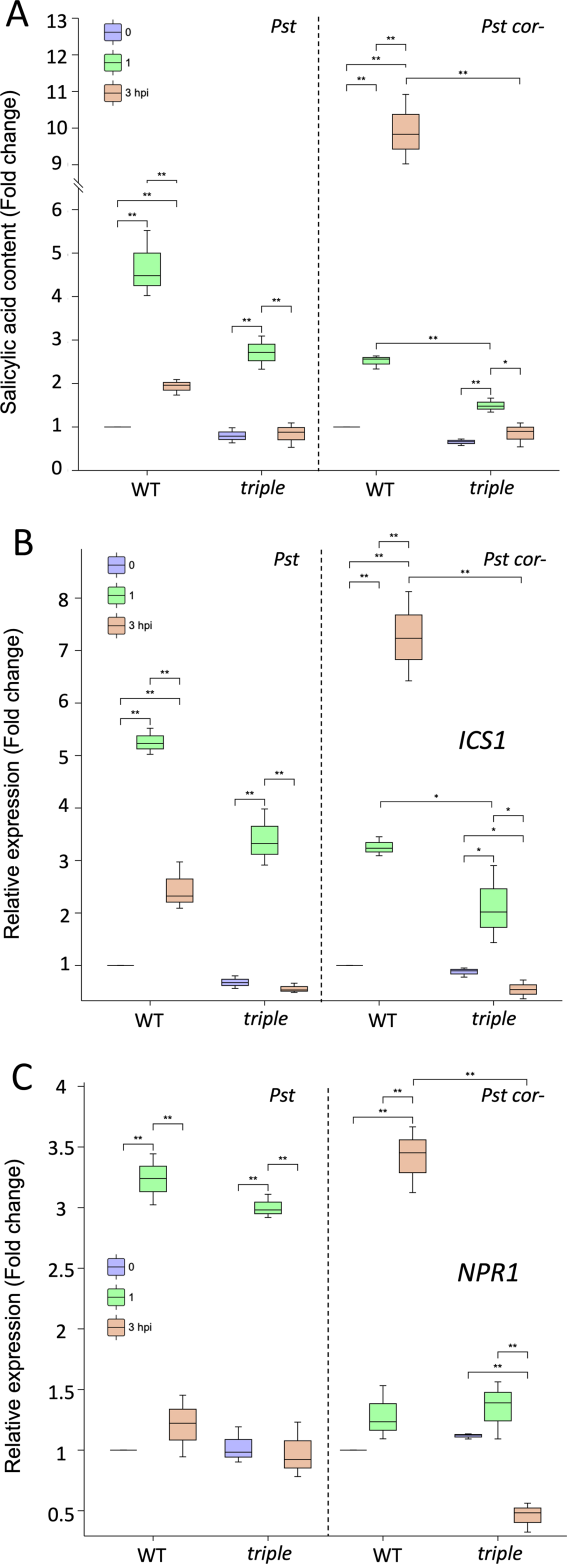
Effect of melatonin deficiency on salicylic acid (SA) content and expression of its biosynthesis and receptor genes under treatments of *Pseudomonas syringae* pv tomato (*Pst*) with or without coronatine (COR). **(A)** SA changes in the wild type (WT) and *triple* mutant after *Pst* and *Pst cor^−^* treatment for 0, 1, and 3 h post infection (hpi). **(B, C)** Relative expression changes of isochorismate synthase 1 (*ICS1*) of SA biosynthesis and a SA receptor non-expressor of pathogenesis-related 1 (*NPR1*), respectively, were quantified by qRT-PCR at different timepoints of *Pst* and *Pst cor^−^* treatments. Error bars indicate the standard deviation, and asterisks represent significant changes by Student’s t-test (* and ** indicating p values < 0.05 and < 0.01, respectively).

In WT treated with either *Pst* or *Pst cor*^−^, the levels of JA-Ile (active form of JA) were repressed at 1 hpi with stomatal closure and was not significantly recovered at 3 hpi (**Figure 4**), and *Pst cor* reopened the stomata (**Figure 2B**). In the case of *Pst cor*^−^, the levels of JA-Ile decreased at both 1 and 3 hpi (**Figure 4**), and stomata remain closed at 3 hpi (**Figure 2C**), consistent with melatonin’s function in stomatal closure (Li et al., 2014). Interestingly, in the *triple* mutant, the contents of JA-Ile were concomitantly increased, particularly in *Pst cor*^−^ compared with that in *Pst* treatment. This result suggests that melatonin enhanced immunity through inhibiting JA biosynthesis. Without melatonin, stomatal reopening was induced by both JA-Ile and/or COR at 3 hpi under *Pst* treatment. However, the JA-Ile content in *triple* was highly accumulated at 3 hpi in *Pst cor*^−^ compared with *Pst* (**Figure 4B**). It indicates that the activated JA signaling pathway at 3 hpi would feedback-regulate linolenic acid metabolism, promoting stomatal reopening even without COR. Not only the inhibition of JA regulated by melatonin but also the promotion of SA (**Figure 5A**) was shown to correlate with the stomatal movement pattern in stomatal immunity (Ou et al., 2022; Meddya et al., 2023; Li et al., 2025). A key enzyme isochorismate synthase 1 (*ICS1*) of SA biosynthesis and an SA receptor non-expressor of pathogenesis-related 1 (*NPR1*) were activated rapidly by melatonin after bacterial recognition and reached the highest level of expression at 3 hpi (**Figures 5B, C**). Clearly, melatonin participates in regulating the JA–SA balanced signaling pathway in stomatal immunity and it enhances immunity by predominantly promoting SA biosynthesis and inhibiting JA biosynthesis.

**Figure 6:**
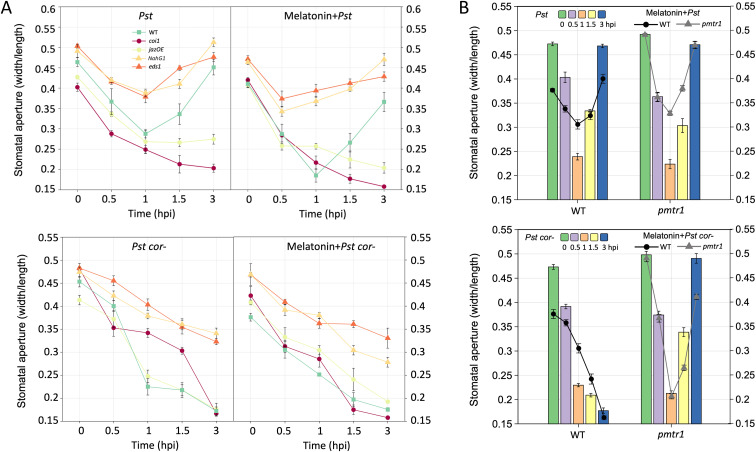
Involvement of jasmonic acid (JA) and salicylic acid (SA) pathways and a melatonin receptor in melatonin-mediated phytohormone crosstalk in stomatal immunity. **(A)** Reverse genetics results showing the roles of JA and SA signaling pathways in stomatal immune response in the presence of COR (upper panel) and without COR (lower panel). *coi1*: COI-deficient mutant; *jazOE*, *JAZ7* overexpression mutant; *NahG1*, SA deficient, and *eds1*, SA-biosynthesis mutant. **(B)** Stomatal movement without melatonin receptor PMTR1 under bacterial and melatonin treatments. The stomatal movement assay was performed five times with three stomata observed each time (n=15).

To validate the above results, different mutants in the hormone pathways were obtained and treated with *Pst* and *Pst cor^−^*. As mentioned earlier, JA-Ile is recognized by the JAZ–COI1 complex which leads to degradation of the transcriptional repressor JAZ7, thereby activating MYC2-mediated JA-responsive gene expression (Liu et al., 2021a). Mutation of *COI1* (JA-Ile and COR receptor) or JAZ overexpression (OE) disrupted stomatal reopening in the presence of COR, and the stomatal aperture was even more decreased by exogenous melatonin (**Figure 6A** top panel). In the absence of COR, stomata showed a continuous closing trend in the 3-h period and no reopening in the WT and the mutants (**Figure 6A** bottom panel).

In SA metabolism, a bacterial salicylate hydroxylase (NahG) can deplete plant SA levels by converting it into catechol (Abreu and Munné-Bosch, 2009). Enhanced Disease Susceptibility 1 (EDS1) is important for SA biosynthesis (Falk et al., 1999). Thus, *NahG* transgenic *Arabidopsis* is deficient in SA and *eds1* has very low SA. Stomata of *NahG* and *eds1* still show the trend of closure at 1 hpi and reopening at 3 hpi, but they maintain a much larger aperture throughout the different time points. With melatonin, stomata closed earlier at 0.5 hpi and then reopened with *Pst* treatment (**Figure 6A** top panel). Without COR, stomata displayed the same trend of closing in WT and JA mutants (**Figure 6A** bottom panel). Please note that irrespective of COR or not, the SA mutants showed much larger apertures than the WT and JA mutants (**Figure 6A**), highlighting the important role of SA in mainlining stomatal immunity. PMTR1 was identified as the first phytomelatonin receptor involved in stomatal closure in *Arabidopsis* (Wei et al., 2018). As expected, the *pmtr1* mutant showed a similar stomatal response as the *triple*; this response could not be remedied with melatonin addition (**Figure 6B**). Clearly, PMTR1 is an effective receptor involved in melatonin-regulated stomatal immunity.

## 4 Discussion

### 4.1 Melatonin is a positive regulator of stomatal immunity

The reduction in disease symptoms and bacterial proliferation is shown in melatonin-pretreated plants, which supports the notion that melatonin is a master hormone regulator of stress responses (Falk et al., 1999; Gimenez-Ibanez et al., 2017; Cheng et al., 2018). The positive regulation of melatonin was observed in stomatal immune responses to both *Pst* and *Pst cor*^−^ (**Figure 1D**, **Supplementary Figure 1C**). The melatonin regulation of stomatal movement has been reported (Wei et al., 2018; Yang et al., 2021). However, only with the *triple* mutant, which significantly decreased endogenous melatonin levels (**Figure 1C**), can we point to a role of melatonin for alleviating bacteria invasion. Although the *triple* mutant showed slightly developmental delay compared with the WT (**Supplementary Figure 1**), the stomatal size, aperture, and movement range are similar to the WT (**Figure 2B**). Additionally, stomatal assays were conducted rapidly using epidermal peels within a short timeframe (Melotto et al., 2006). Furthermore, in melatonin-related mutants, defense signaling pathways operate as a functionally independent module (Lee et al., 2014). Thus, it is highly unlikely that the stomatal movement phenotype of the *triple* mutant can be attributed to developmental delay. Instead, it only stands out when COR is deficient (**Figure 2C**).

It has been shown that not only PTI but also ETI is rapidly enhanced by melatonin to modulate nucleotide-binding and receptor functions that synergize PTI and ETI defense (Tiwari et al., 2022). The priming effect of melatonin is crucial for the first line of defense to decrease bacterial entry (**Supplementary Figure 1C**). This result has been corroborated with improved disease outcomes in previous studies (Mandal et al., 2018; Zhu et al., 2021b; Yang et al., 2023). Although this study is focused on the early stage of stomatal defense, future studies may extend the time course and include apoplastic defense. Additionally, melatonin is well known to have circadian hormone roles in both animals and plants (Wei et al., 2022; Mamun et al., 2023; Bargunam et al., 2025). Future research may also investigate how this immunity role plays out in the context of circadian regulation.

### 4.2 Melatonin modulates linolenic acid metabolism, COR/JA, and SA cross talk in stomatal immunity

**Figure 7:**
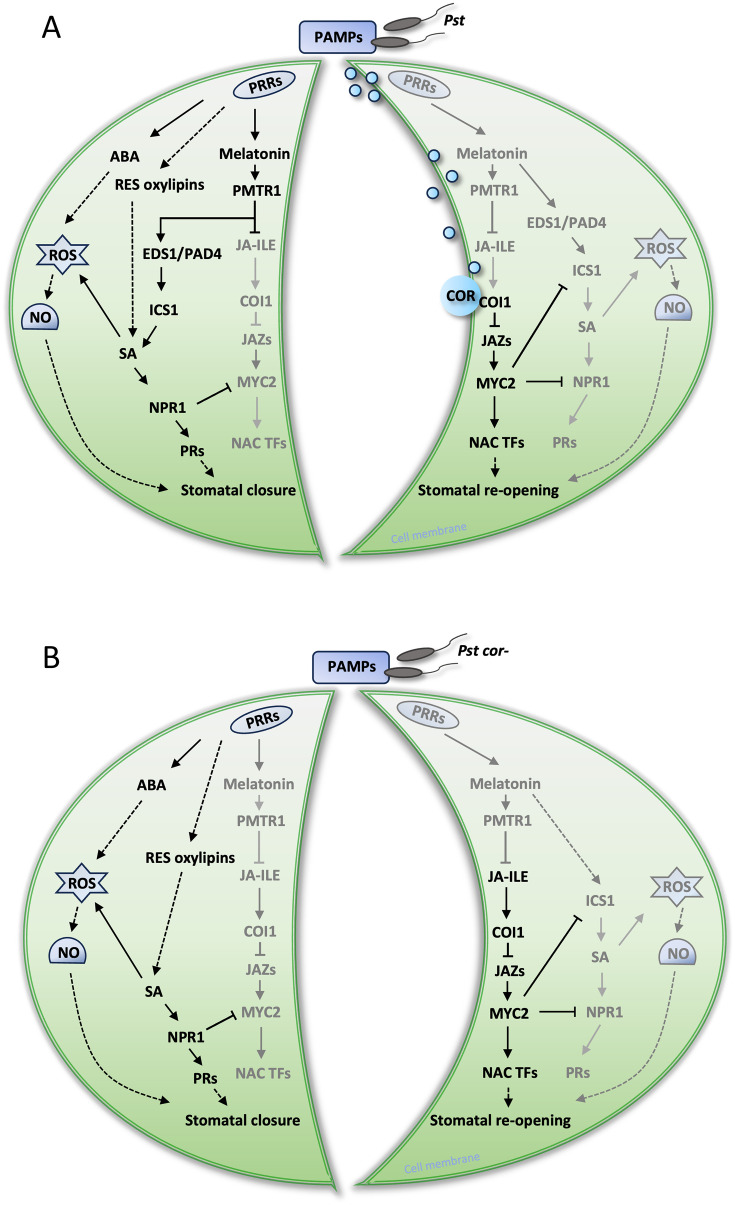
Proposed model depicting melatonin-mediated phytohormone balancing network under bacteria-induced stomatal immunity. **(A)** Stomatal closure at 1 h post infection (hpi) (left guard cell) and reopening at 3 hpi (right guard cell) under *Pst* treatment. When the pattern recognition receptors (PRRs) recognize pathogen-associated molecular patterns (PAMPs), melatonin functions via its receptor PMTR1 to inhibit JA signaling and activate SA signaling. Together with ABA signaling, ROS burst, and NO regulation, stomata are closed. At 3 hpi, the bacterial effector COR hijacks the core module of JA (COI1-JAZs-TFs), inhibits melatonin and SA-NPR1 functions, and activates transcription factors (TFs) like NACs, leading to stomatal reopening. **(B)** Stomatal closure at 1 hpi (left guard cell) and reopening at 3 hpi (right guard cell) without melatonin under *Pst cor−* treatment. In the *triple* mutant, stomata are closed through ABA-dependent and/or independent pathways. In ABA-dependent signaling, ABA-mediated ROS and NO production causes stomata to close. In the ABA-independent pathway, RES oxylipins function through the SA-NPR1 immunity pathway to close the stomata. At 3 hpi, JA biosynthesis is increased due to lack of melatonin suppression. The core module of JA-Ile (COI1-JAZs-TFs) is activated to inhibit SA-NPR1 and reopen the stomata. The integrated network output of melatonin regulates stomatal aperture through multi-hormone cross talk in the first line of defense against pathogen invasion. Solid and dotted lines indicate direct and indirect connections, respectively.

In counteracting the stomatal innate immunity, certain bacterial pathogens (e.g., *Pst* DC3000) evolved COR to reopen stomata and facilitate bacterial entry (Melotto et al., 2006, 2008; Pang et al., 2020). In our study, a surprising finding was revealed with the melatonin mutant treated with *Pst cor^−^*, in which stomata reopened without COR (**Figure 2C**). However, the presence/absence or addition of melatonin did not appear to make a difference (**Figures 1D**, **2B**), implying a dispensable role of melatonin in stomatal immunity in the presence of COR. COR is known to reopen the initially closed stomata through hijacking the JA signaling pathway by binding to the JA receptor COI1 with high affinity (Hayashi et al., 2023; Pavlovič et al., 2024) (**Figure 7**), suppressing SA (**Figures 5**, **6A**) (Liu et al., 2021b; Sun et al., 2021). Interestingly, in tomato cold response, melatonin acts synergistically with JA to form a positive feedback loop (Sheard et al., 2010; Ding et al., 2021). Melatonin seems to be adequate to enhance SA-based immunity as previously reported (Liu et al., 2023; Li et al., 2025), through altering the JA–SA antagonism in stomatal immunity (Zhu et al., 2024). First, melatonin suppresses linolenic acid metabolism, which provides pivotal starting materials for JA biosynthesis (Roychowdhury et al., 2024) (**Figure 4B**). Second, SA-induced stomatal closure and ROS production are dependent on melatonin signaling (Li et al., 2025). Third, melatonin is involved in NPR1 stabilization, a key integrator of SA signaling that suppresses JA-responsive genes (Li et al., 2025). Therefore, melatonin can tilt the JA–SA antagonistic balance toward SA, thereby promoting stomatal closure. Without melatonin, the balance tilts toward JA signaling, thereby promoting a stomatal opening event in the absence of COR (**Figures 2C**, **5**, **7**). Interestingly, melatonin was shown to synergistically enhance JA signaling to promote defense when plants were attacked by necrotrophic fungi or herbivores (Liu et al., 2019; Zhu et al., 2021b; Yan et al., 2025). This apparent contradiction with bacterial defense highlights that the immune function of melatonin is contextual, likely depending on the initial recognition and signaling (Shan et al., 2025). Alternatively, melatonin could be involved in scavenging COR-induced ROS and thereby facilitate stomatal reopening (Moreno and Campos, 2022; Uddin et al., 2022). This role may be insignificant as in the t*riple* mutant stomata reopen normally (**Figure 2B**). Moreover, studies have shown that in WT *Arabidopsis*, melatonin increased endogenous SA and ABA levels in defense against *Pst* DC3000 (Liu et al., 2023), indicating the synergistic relationship among ABA, SA, and melatonin.

### 4.3 Melatonin regulated hormone crosstalk in stomatal defense through PMTR1 and ROS/NO

PMTR1 is the first plant melatonin receptor identified in *A. thaliana* to regulate stomatal closure (Wei et al., 2018) and immune response (Yang et al., 2021). The *pmtr1* mutant showed a similar stomatal response as the melatonin mutant and both compromised immunity against bacterial pathogens (**Figure 6B**). With no response of melatonin addition, it is suggested that PMTR1 may have a notable regulatory node in maintaining defense hormone responses in stomatal immunity. PMTR1 was placed at the upstream of a signaling cascade that integrates ROS, NO, and hormone cross talk to enforce stomatal closure (Wang et al., 2023). ROS are known to be involved in melatonin-mediated stomatal immunity and stress responses (Wang et al., 2023; Xiao et al., 2023; Ahammed et al., 2024). In our study, multiomics revealed involvement of ROS metabolism in melatonin-regulated stomatal immunity, e.g., through enrichment of ascorbate, aldarate, and the pentose phosphate pathways (PPP) (**Figure 5A**). Ascorbate, aldarate, and PPP all protect cells from oxidative stress. The PPP produces a critical electron donor NADPH that fuels the cellular antioxidant systems (e.g., the ascorbate–glutathione cycle) (Wu et al., 2023). In addition, melatonin increases NO biosynthesis by activating key enzymes like nitric oxide synthase and nitrate reductase. The boost in NO production is crucial as it plays a role in decreasing stomatal conductance (Ghorbani et al., 2023; Lee et al., 2025). However, NO also increases melatonin biosynthesis and its signaling under biotic stress conditions (Ghorbani et al., 2023). As reflected by the omics data, the metabolic pathways related to NO accumulation were enriched, including arginine and proline metabolism, glutathione metabolism, alanine/aspartate, and glutamate metabolism (**Supplementary Table 2**, **Supplementary Figure 3**). It is suggested that NO acts downstream of melatonin in tomato stomatal immunity (Zhu et al., 2019). ABA signaling in guard cells is known to involve ROS and NO, which cause cellular redox changes (Raschke, 1987; Pei et al., 1998; Bharath et al., 2021). Upon pathogen infection, melatonin was found to promote stomatal closure by inducing ABA signaling and actively blocks pathogen invasion (Zhao et al., 2021). The interplay between ROS/NO and different hormones, including melatonin, ABA, JA, and SA further complicate the cellular regulatory networks underlying stomatal defense. It is likely that the different hormone actions converge at ROS and NO, leading to upstream and/or downstream redox modifications to key nodes of molecular networks. Characterizing the redox “codes” using modern proteomics technologies (Parker et al., 2015; Yu et al., 2020; Balmant et al., 2021) may help understand the dynamic process of plant’s first line of defense against pathogens.

## 5 Conclusion

In this study, we aimed to elucidate the regulatory role and underlying molecular mechanisms of melatonin in plant stomatal immunity against *Pseudomonas syringae* infection. By investigating a melatonin-deficient mutant, we demonstrated that melatonin plays a pivotal role in maintaining stomatal closure, effectively counteracting bacterial invasion regardless of the presence of COR. Integrated metabolomic and proteomic analyses further revealed that melatonin enhances plant defense against bacteria by modulating JA–SA antagonistic coordination, while maintaining ROS/NO homeostasis during stomatal movement to fine-tune plant defense responses. The schematic representation of melatonin-mediated multi-hormone crosstalk in stomatal immunity (**Figure 7**) provides a comprehensive understanding of the molecular underpinnings of guard cell-mediated innate immunity. These findings not only clarify how melatonin coordinates early defense responses but also identify potential molecular targets for marker-based breeding to enhance disease resistance in crops.

## Data Availability

The raw data of metabolomics and proteomics are available through Zenodo repository DOI: 10.5281/zenodo.17553628.

## Supplementary material

Supplementary Figure 1 Genotypes and phenotypes of WT and *triple* mutant after bacterial spray inoculation. **(A)** Schematic diagram of developing the melatonin biosynthesis *triple*- mutant using three homozygous single mutants: *comt1*, caffeic acid O-methyltransferase 1; *snat1*, serotonin N-acetyltransferase 1; and *asmt1*, acetylserotonin O-Methyltransferase 1. LB, T-DNA left border; RP, gene right primer at 3’ end; and LP, gene left primer at 5’ end (please see **Supplementary Table 1**). (**B**) Phenotypic comparison between WT and *triple* plants at the seedling stage (left panel) and bolting stage (right panel). Bar=1 cm. (**C**) Disease symptoms of WT and *triple* leaves two days after the bacterial spray inoculation. Bar=1 cm.

Supplementary Figure 2 Proteomic profiling of guard cell proteins changed in response to treatments with *Pseudomonas syringae* pv tomato (*Pst*) and *Pst* without coronatine (*Pst cor^-^*). **(A)** Upset plot illustrating the interactions of differentially abundant proteins (DAPs) identified across the comparison groups. Each vertical bar represents the number of protein intersections, indicated by the connected dots below. The top horizontal bar plot shows the total number of DAPs in each group. **(B)** Gene Ontology (GO) categories of DAPs under the bacterial treatments. The bar chart shows the top significantly enriched GO terms categorized into Cellular Component (CC), Biological Process (BP), and Molecular Function (MF). The heatmap represents the DAPs between the *triple* and WT groups under each treatment (*p*-value<0.05, fold change cut-off was set to <0.5 or >2).

Supplementary Figure 3 KEGG pathway analysis of significantly changed metabolites (SCMs) related to melatonin in stomatal response to treatments with *Pseudomonas syringae* pv tomato (*Pst*) and *Pst* without coronatine (*Pst cor^-^*). Based on KEGG database, the heatmap represents the overlapped pathway of SCMs among six treatments between the *triple* and WT groups (*p*-value<0.05, fold change cut-off was set to <0.5 or >2).

Supplementary Table 2 Multiple comparison analyses of metabolites under pathogenic treatment at different time points.

Supplementary Table 3 Multiple comparison analyses of differentially abundant proteins (DAPs) and their functional annotations.
