# Supplementary figures and images for: Melatonin promotes stomatal immunity by mediating phytohormone crosstalk in Arabidopsis thaliana
Source: Front Plant Sci. 2026 Mar 24;17:1801537. doi: 10.3389/fpls.2026.1801537 (PMC13053500; doi:10.3389/fpls.2026.1801537)

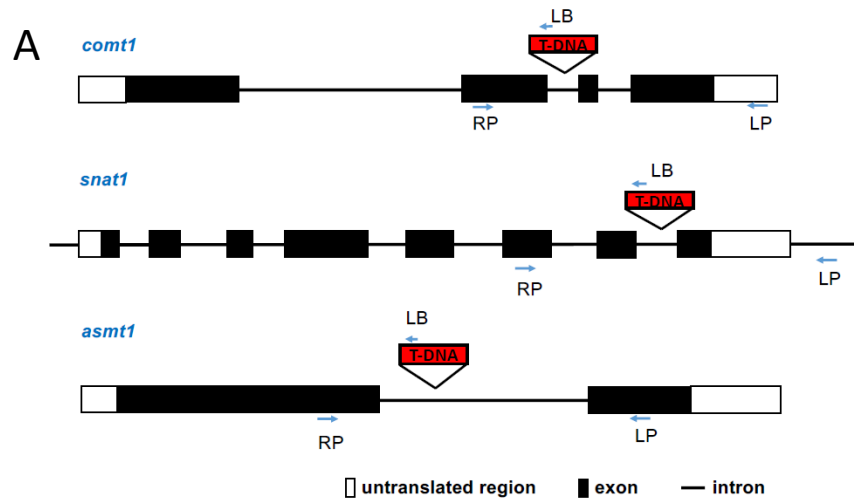

**B**

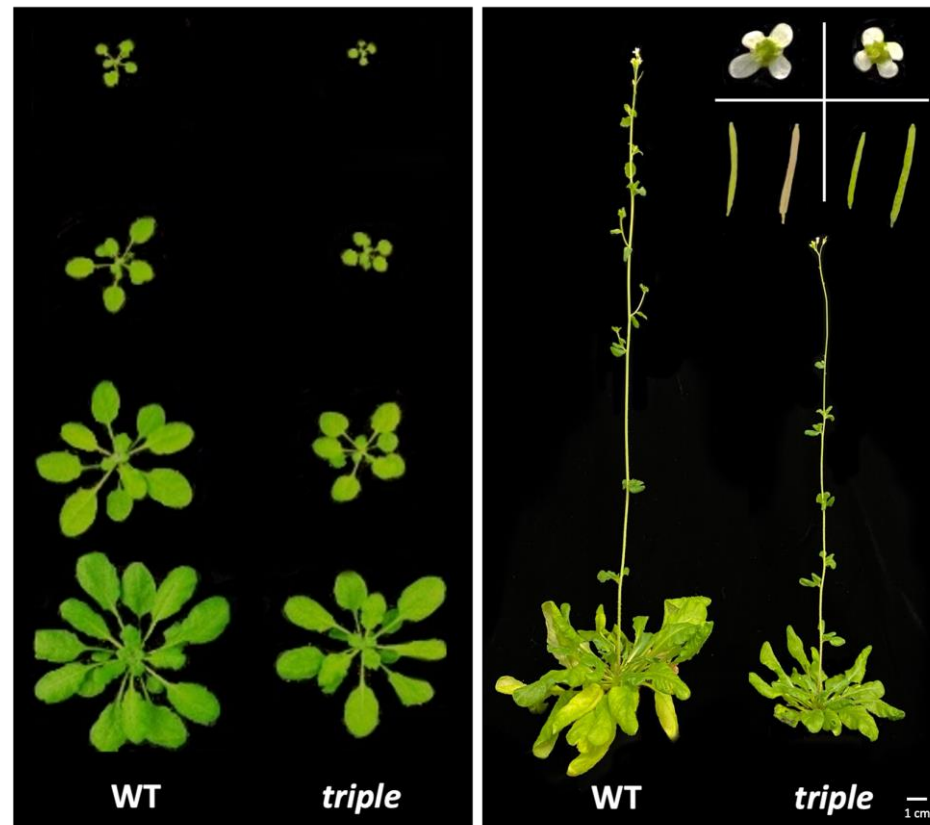

**C**

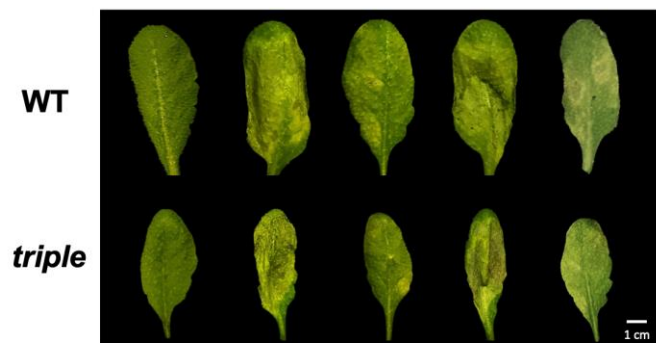

|                            |   |   |   |   |   |
|----------------------------|---|---|---|---|---|
| Melatonin                  | - | - | + | - | + |
| <i>Pst</i>                 | - | + | + | - | - |
| <i>Pst cor<sup>-</sup></i> | - | - | - | + | + |

Figure S1

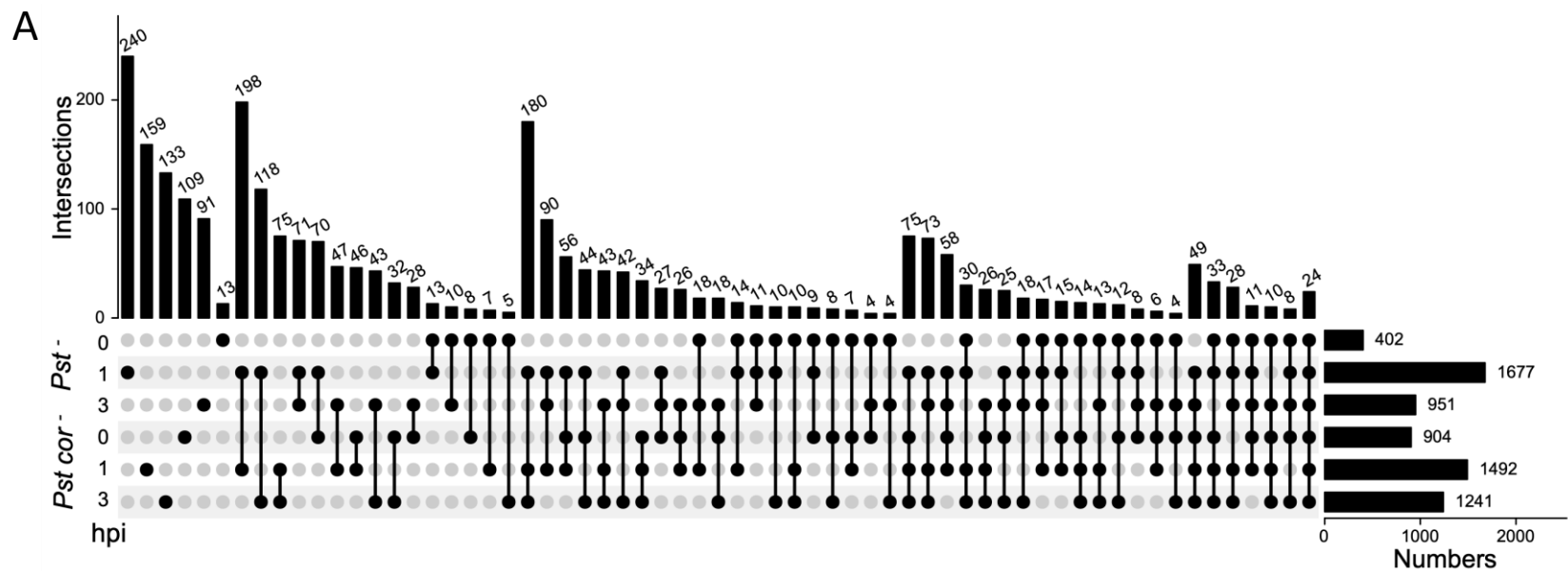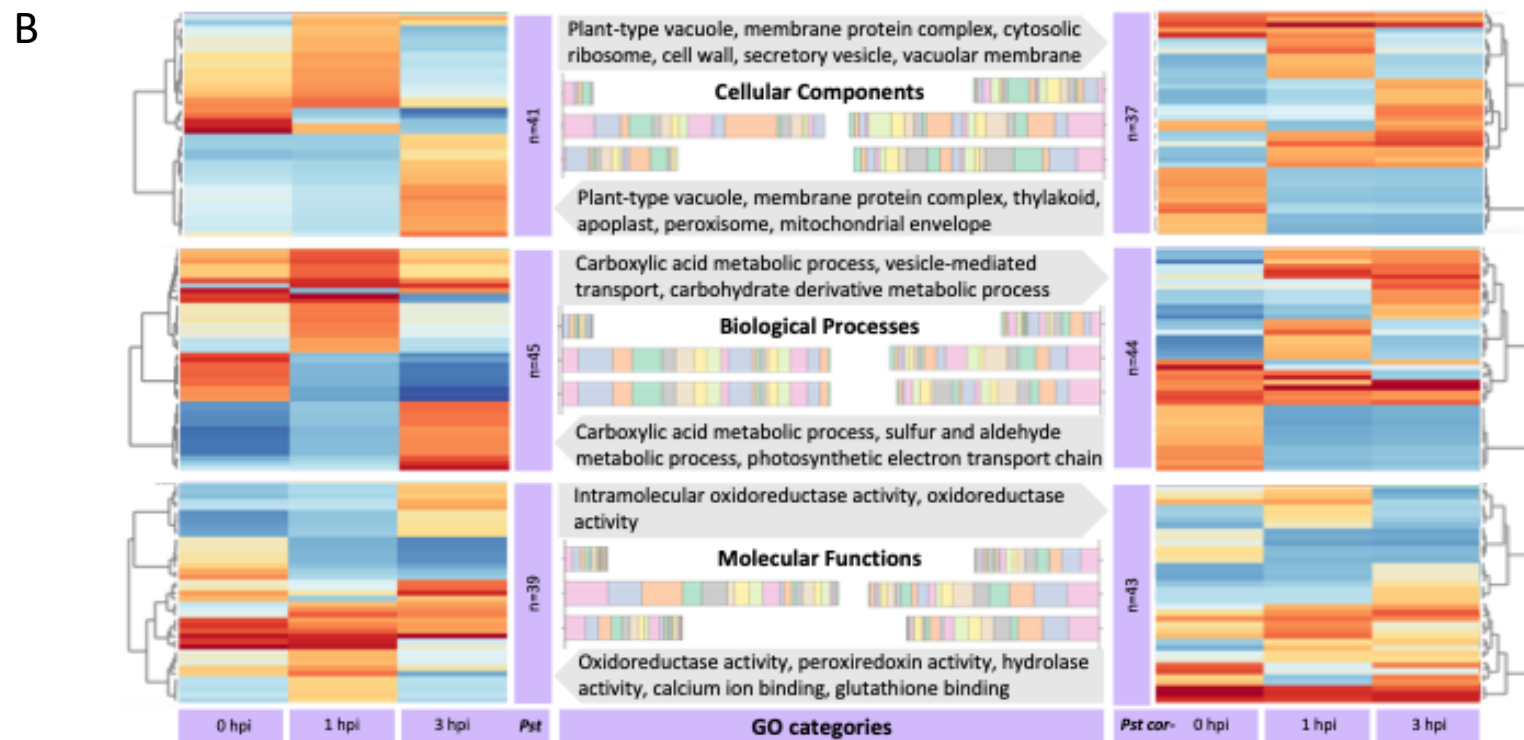

Figure S2

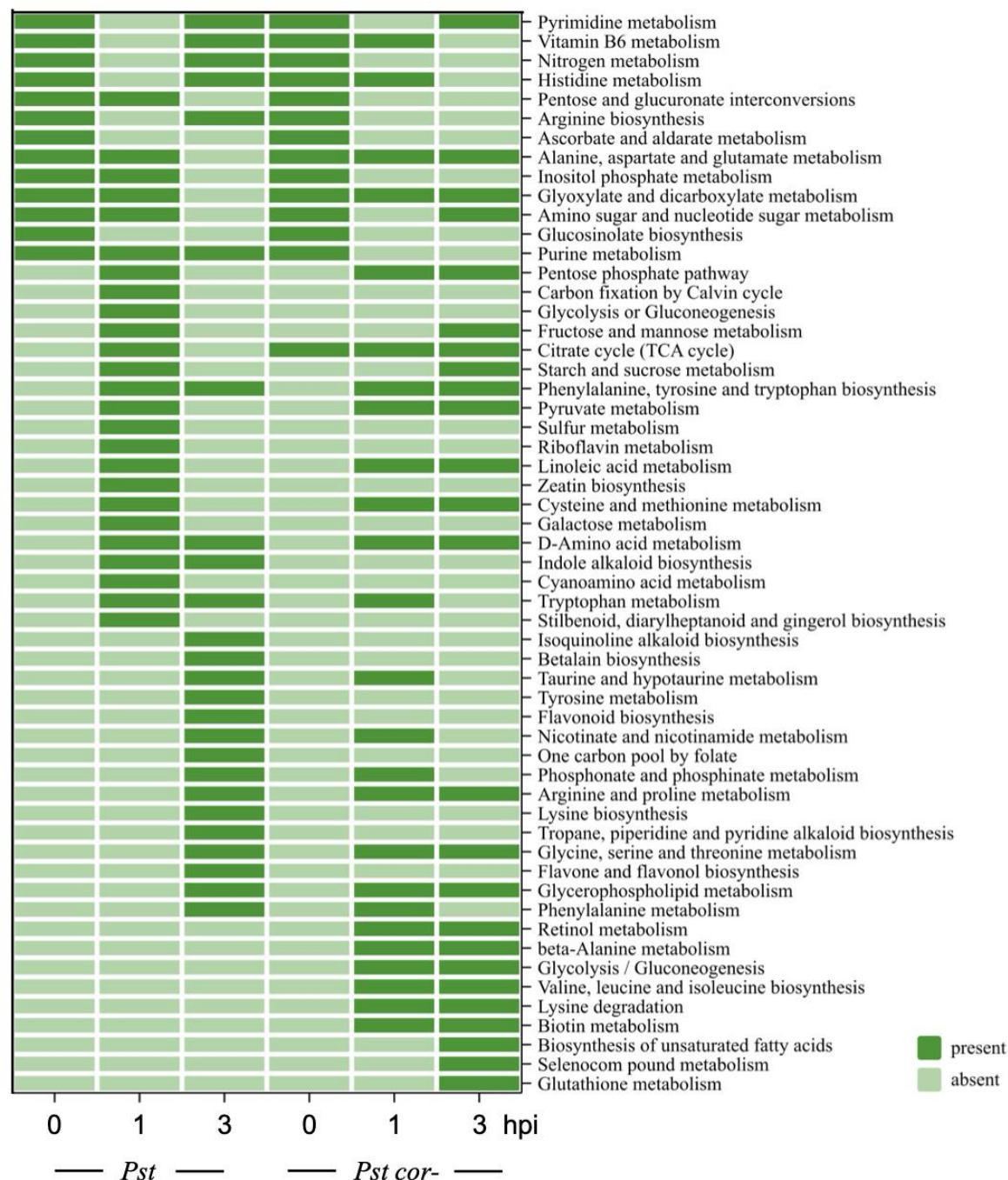

Figure S3

Supplement: Supplementary Figure 1 — Genotypes and phenotypes of WT and triple mutant after bacterial spray inoculation. (A) Schematic diagram of developing the melatonin biosynthesis triple- mutant using three homozygous single mutants: comt1, caffeic acid O-methyltransferase 1; snat1, serotonin N-acetyltransferase 1; and asmt1, acetylserotonin O-Methyltransferase 1. LB, T-DNA left border; RP, gene right primer at 3’ end; and LP, gene left primer at 5’ end (please see Supplementary Table 1). (B) Phenotypic comparison between WT and triple plants at the seedling stage (left panel) and bolting stage (right panel). Bar=1 cm. (C) Disease symptoms of WT and triple leaves two days after the bacterial spray inoculation. Bar=1 cm. [file DataSheet1.pdf]
